# Calculation of differential pulmonary blood flow in patients with congenital heart disease using pulmonary artery flow versus pulmonary vein flow

**DOI:** 10.1186/1532-429X-18-S1-P181

**Published:** 2016-01-27

**Authors:** Nao Sasaki, Jun Sasaki, Juan Carlos G Muniz

**Affiliations:** Pediatric Cardiology, Nicklaus Children's Hospital, Miami, FL USA

## Background

Phase contrast magnetic resonance (PCMR) has been shown to provide accurate measurement of differential branch pulmonary artery (BPA) flow in patients with congenital heart disease when compared to the gold standard, lung perfusion scintigraphy (LPS). Although most studies have compared the ratio of net BPA flow volume (NFV) to LPS, one study found that forward flow volume (FFV) correlated more accurately. PCMR quantification of pulmonary venous (PV) flow has also been shown to be feasible and highly reproducible. We sought to calculate differential pulmonary blood flow using PV flow and compare the results with both NFV and FFV calculations.

## Methods

Retrospective review of 56 patients with congenital heart disease who had phased contrast flow quantification of both branch pulmonary arteries and pulmonary veins during the same study. FFV and NFV of RPA and LPA, as well as their ratios were calculated and were compared to RPV and LPV flow volume.

## Results

There were 22 males, and the median age was 12.2 years of age. 37 patients had tetralogy of Fallot and the remainder had other conotruncal abnormalities requiring intervention to the right ventricular outflow tract. Agreement between NFV and PV was superior (intraclass correlation; Ri=0.954 right, Ri=0.857 left, p < 0.001) than FFV and PV (Ri=0.630 right, Ri=0.615 left, p < 0.001). The BPA flow ratio calculated by both NFV and FFV had good agreement with PV flow ratio (Ri=0.904 p < 0.001 and Ri=0.860 p < 0.001, respectively).

## Conclusions

Differential lung perfusion calculated using the PV flow method has excellent agreement with that calculated using PA flow measurements. This technique may be useful in patients where accurate measurement of BPA flow may not be feasible (e.g., those with significant stenoses or stents).

**Figure 1:**
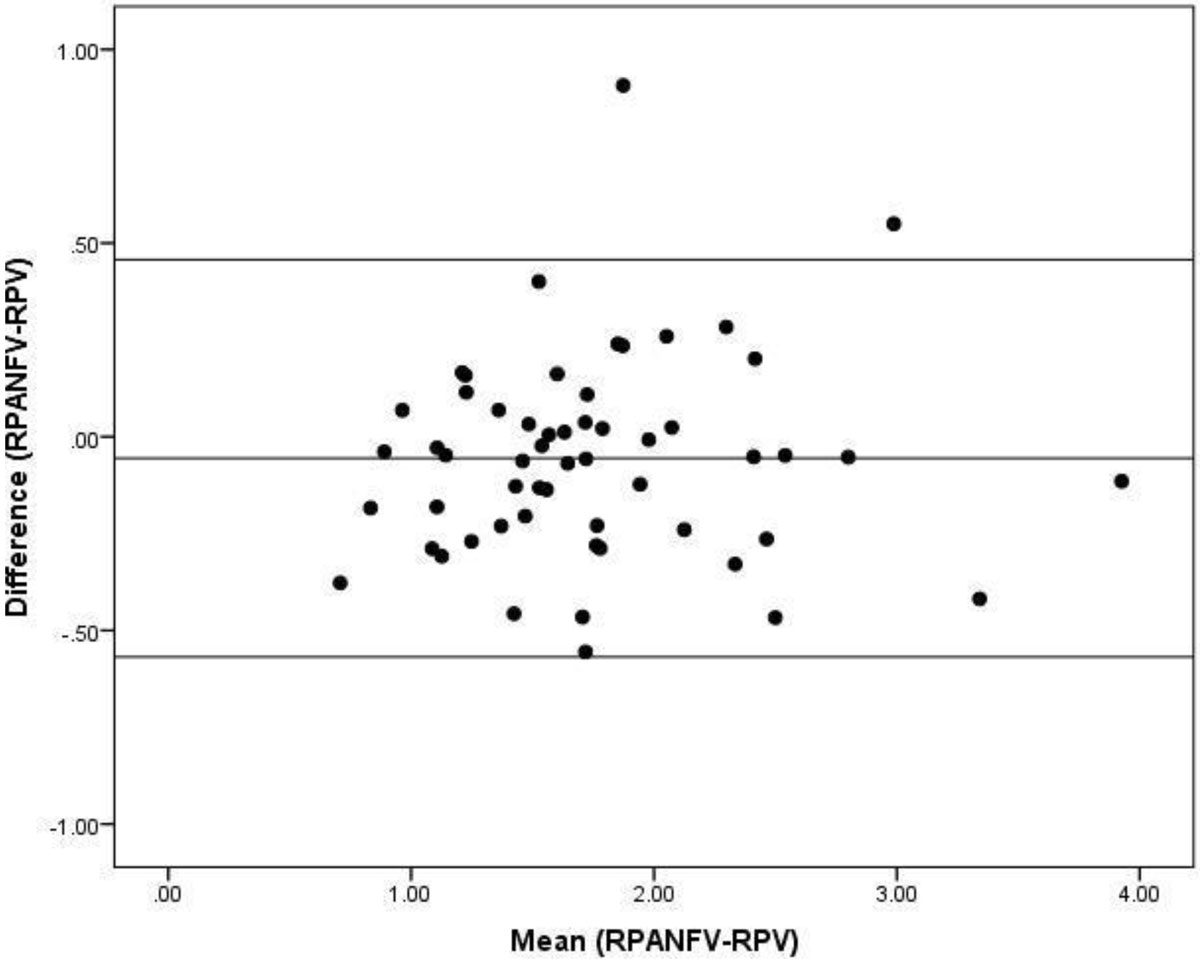
Figure 1

**Figure 2:**
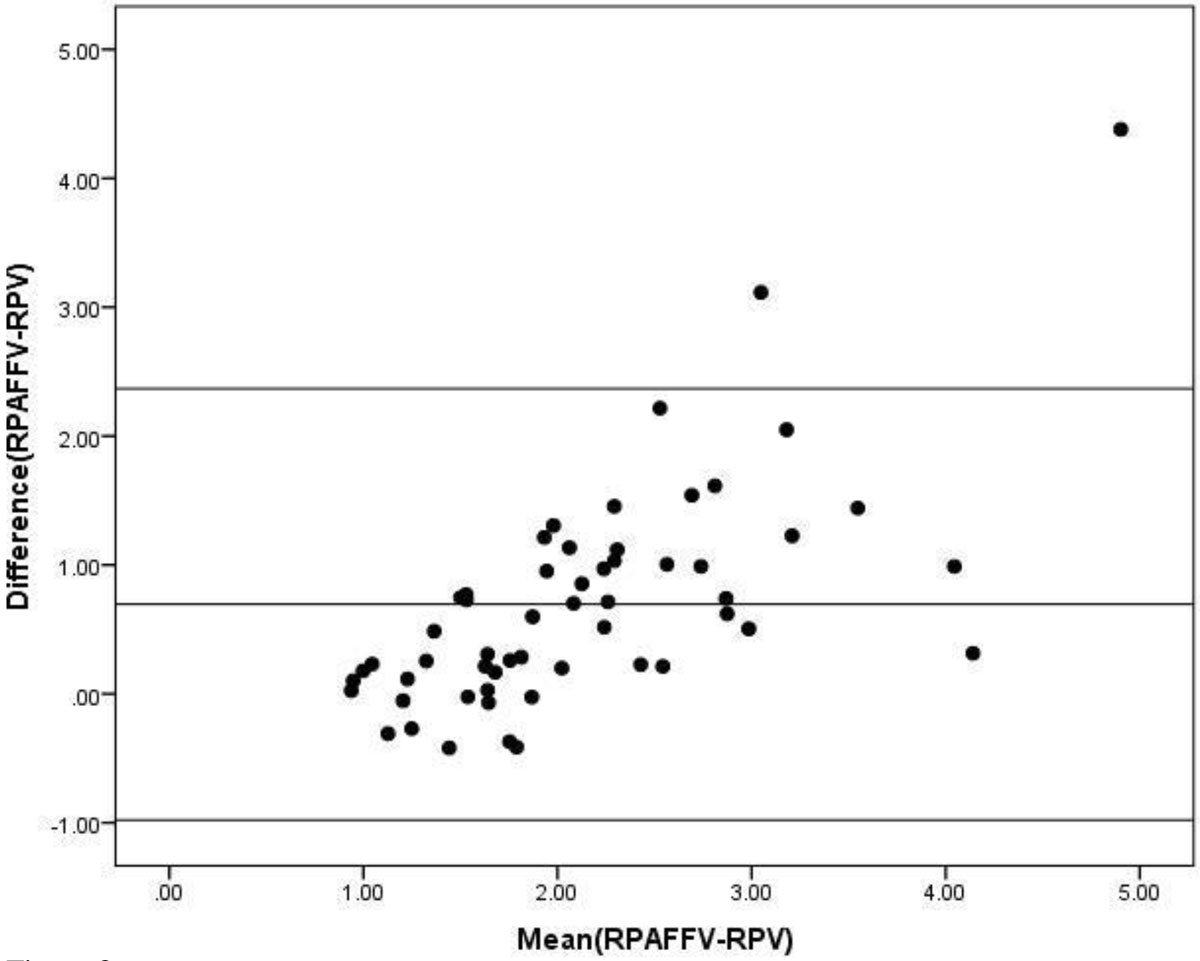
Figure 2

